# Supplementary material for: Time-series transcriptomic analysis reveals novel gene modules that control theanine biosynthesis in tea plant (Camellia sinensis)
Source: PLoS One. 2020 Sep 10;15(9):e0238175. doi: 10.1371/journal.pone.0238175 (PMC7482930; doi:10.1371/journal.pone.0238175)

**S4 Fig. Volcano plot that denoted differential expressed genes (DEGs) in EA activation.** The red, blue, and black points represented upregulated genes, downregulated genes, and non-significant-differential-expressed genes, respectively. 1d, 3d, 6d, 9d, 12d, 18d, and 24d in (a)-(g) denoted the corresponding time points of 1, 3, 6, 9, 12, 18, 24 day(s) of EA treatment. The log_2_-Counts Per Million (log CPM) was used to transform raw counts onto a scale and conduct gene differential expression analysis using the *cpm* and *exactTest* function in edgeR.


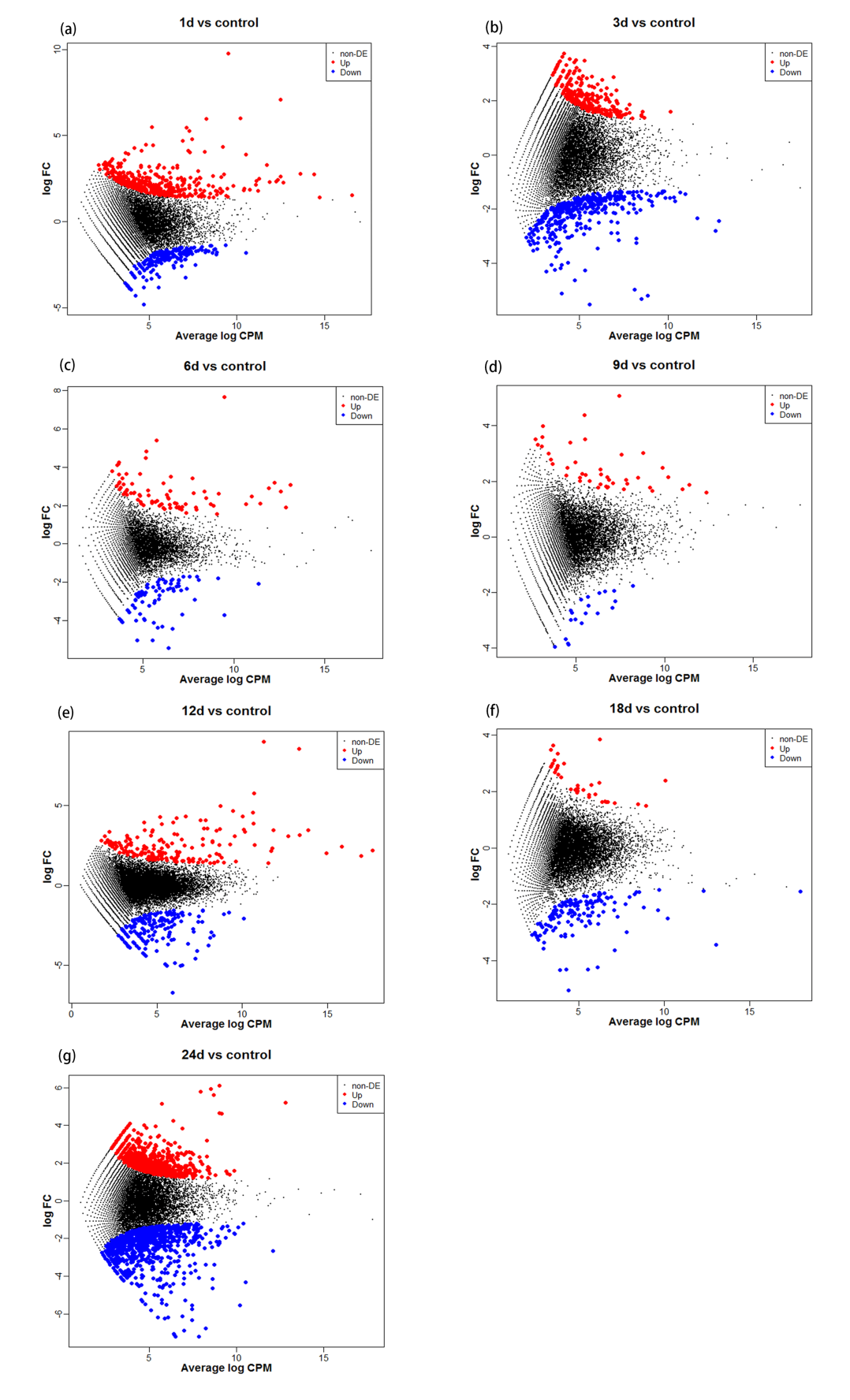

Supplement: S1 Fig — (DOCX) [file pone.0238175.s004.docx]
