# Supplementary figures and images for: Time-series transcriptomic analysis reveals novel gene modules that control theanine biosynthesis in tea plant (Camellia sinensis)
Source: PLoS One. 2020 Sep 10;15(9):e0238175. doi: 10.1371/journal.pone.0238175 (PMC7482930; doi:10.1371/journal.pone.0238175)

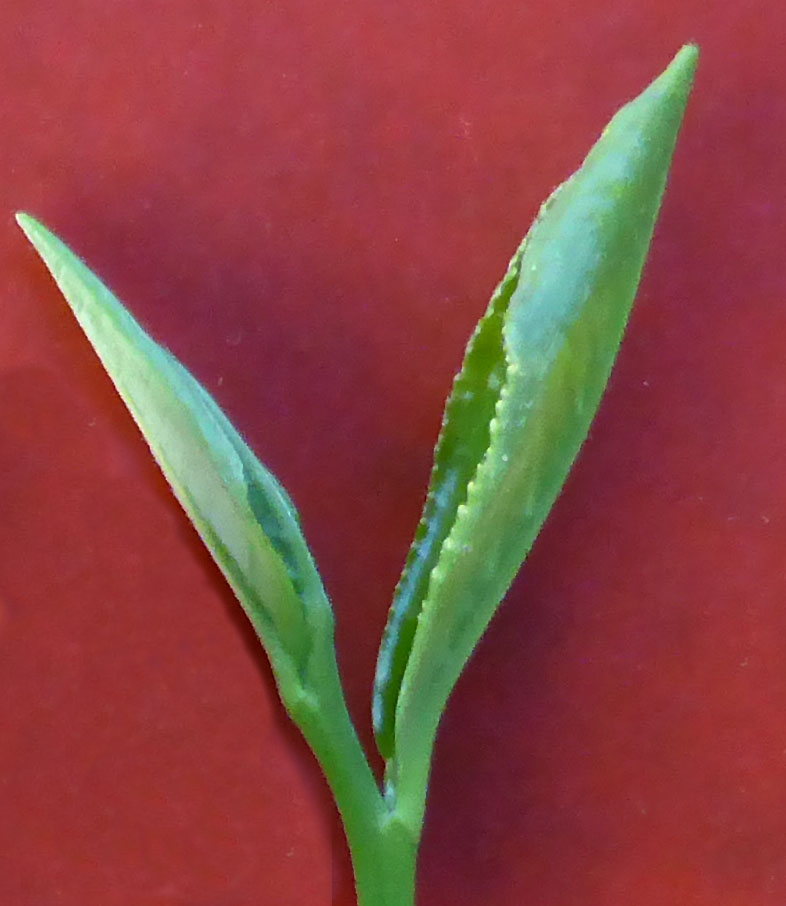

Supplement: S2 Fig — (TIF) [file pone.0238175.s005.tif]
